# A Push–Pull Mechanism Between PRRT2 and β4-subunit Differentially Regulates Membrane Exposure and Biophysical Properties of NaV1.2 Sodium Channels

**DOI:** 10.1007/s12035-022-03112-x

**Published:** 2022-11-28

**Authors:** Pierluigi Valente, Antonella Marte, Francesca Franchi, Bruno Sterlini, Silvia Casagrande, Anna Corradi, Pietro Baldelli, Fabio Benfenati

**Affiliations:** 1 Department of Experimental Medicine, Section of Physiology, University of Genova, Viale Benedetto XV, 3, 16132 Genova, Italy; 2 IRCCS, Ospedale Policlinico San Martino, Largo Rosanna Benzi 10, 16132 Genova, Italy; 3 Center for Synaptic Neuroscience and Technology, Istituto Italiano Di Tecnologia, Largo Rosanna Benzi 10, 16132 Genova, Italy

**Keywords:** Proline-rich transmembrane protein 2, Intrinsic excitability, Transient Na^+^ current, Persistent Na^+^ current, Resurgent Na^+^ current

## Abstract

Proline-rich transmembrane protein 2 (PRRT2) is a neuron-specific protein implicated in the control of neurotransmitter release and neural network stability. Accordingly, PRRT2 loss-of-function mutations associate with pleiotropic paroxysmal neurological disorders, including paroxysmal kinesigenic dyskinesia, episodic ataxia, benign familial infantile seizures, and hemiplegic migraine. PRRT2 is a negative modulator of the membrane exposure and biophysical properties of Na^+^ channels Na_V_1.2/Na_V_1.6 predominantly expressed in brain glutamatergic neurons. Na_V_ channels form complexes with β-subunits that facilitate the membrane targeting and the activation of the α-subunits. The opposite effects of PRRT2 and β-subunits on Na_V_ channels raises the question of whether PRRT2 and β-subunits interact or compete for common binding sites on the α-subunit, generating Na^+^ channel complexes with distinct functional properties. Using a heterologous expression system, we have observed that β-subunits and PRRT2 do not interact with each other and act as independent non-competitive modulators of Na_V_1.2 channel trafficking and biophysical properties. PRRT2 antagonizes the β4-induced increase in expression and functional activation of the transient and persistent Na_V_1.2 currents, without affecting resurgent current. The data indicate that β4-subunit and PRRT2 form a push–pull system that finely tunes the membrane expression and function of Na_V_ channels and the intrinsic neuronal excitability.

**Supplementary Information:**

The online version contains supplementary material available at 10.1007/s12035-022-03112-x.

## Introduction

Mutations in the proline-rich transmembrane protein 2 (PRRT2) gene cause a broad and heterogeneous spectrum of neurological diseases sharing a paroxysmal nature, such as paroxysmal kinesigenic dyskinesia, episodic ataxia, benign familial infantile seizures, and hemiplegic migraine [1]. Nonsense, missense, and frameshift mutations have been reported. The vast majority of these mutations (~ 80%) carry the same frameshift single-nucleotide duplication c.649dupC [1–3] that, via mRNA decay or translation of a non-functional truncated protein, give raise to haploinsufficiency [4–7]. The diverse and pleiotropic clinical manifestations of paroxysmal *PRRT2*-linked diseases with no clear genotype–phenotype correlations suggest that the PRRT2 protein has the function of preserving the stability of neuronal networks in which it is expressed [8].

Structurally, PRRT2 is a neuron-specific type 2 integral membrane protein with a large cytosolic N-terminal domain [9], expressed in the axonal and presynaptic domains [10]. At the presynaptic level, PRRT2 orchestrates the Ca^2+^ sensitivity of synaptic vesicle exocytosis and by interacting with the SNARE complex, synaptotagmins 1/2, P/Q-type voltage-gated Ca^2+^ channels (Ca_V_), and the actin cytoskeleton [10–14]. At this level, PRRT2 enhances the probability of release to single stimuli and favors synaptic depression during sustained activity [10, 14, 15].

In addition to regulation of synaptic transmission, an important function of PRRT2 is the control of intrinsic excitability through specific interactions with both voltage-gated Na^+^ (Na_V_) 1.2/1.6 channels and the α1/α3-subunits of Na^+^/K^+^ ATPase [15–18]. The diffuse neuronal network hyperexcitability observed in PRRT2 knockout (KO) mice recapitulates the pathological hallmarks of PRRT2-linked disorders [8, 19–21]. It has been found that PRRT2 exerts an inhibitory constraint on membrane targeting and exposure of Na_V_1.2/Na_V_1.6, the two main Na^+^ channel subtypes expressed in excitatory neurons and modulates their biophysical properties [15–17]. Such observation represents the molecular basis of the reported efficacy of low doses of the Na_V _blocker carbamazepine in ameliorating the PRRT2-linked clinical manifestations [4, 22–25].

Na_V_ channels are embedded in a multicomponent membrane signaling complex that involves various integral membrane proteins [26] in which β-subunits are the prominent members forming Na_V_ heteromeric complexes. These specific supramolecular complexes are composed of a single pore-forming α-subunit, a non-covalently associated β1/β3-subunit, and a covalently linked β2/β4-subunit via a single disulfide bond [27]. β-Subunits present a large extracellular Ig-like domain, a single multifunctional transmembrane segment, and a short cytoplasmic tail allowing them to positively modulate the gating properties, membrane targeting, and expression levels of the α-subunits [28]. The recently characterized negative modulatory effect of PRRT2 on Na_V_1.2/Na_V_1.6, expressed in the absence of β-subunits, raises the question of whether PRRT2 and β-subunits interact or compete for the binding and modulation of Na_V_ targeting and biophysics, generating Na^+^ channel complexes with distinct functional properties.

Using a heterologous expression system, we have observed that β-subunits do not interact directly with PRRT2. Moreover, the β4-subunit and PRRT2 operate as non-competitive modulators of Na_V_1.2 channel trafficking, membrane expression, and biophysical properties. PRRT2 antagonizes both the β4-induced increases in expression of Na_V_1.2 subunits and the functional activation of the transient and persistent Na_V_1.2 currents, without affecting resurgent current. The data indicate that β4-subunit and PRRT2 form a push–pull system that finely tunes the membrane expression and function of Na^+^ channels and that such dual modulation is perturbed by PRRT2 loss-of-function mutation, resulting in hyperactivity of Na^+^ channels and network hyperexcitability.

## Materials and Methods

### Cell Culture and Transfection

HEK293 cells were grown in DMEM-F12 supplemented with 0.5 mM pyruvate 10% fetal bovine serum, 1% L-glutamine, 50 U/ml penicillin, and 50 µg/ml streptomycin and maintained at 37 °C in 5% CO_2_. For HEK293 cells stably expressing human Na_V_1.2 (SCN2A), 500 µg/ml G418 was added to the medium to select of Na_V_ channel expressing cells. All reagents were purchased from Thermo Fisher Scientific. For testing β1-β4-subunit/Na_V_1.2 interactions, Na_V_1.2-expressing HEK293 cells were transfected with either FLAG-tagged β-subunit (*SCN1B* [NM_001037], *SCN2B* [NM_004588], SCN3B [NM_018400], SCN4B [NM_174934]; Origene) or FLAG-tagged bacterial alkaline phosphatase (BAP; Sigma-Aldrich). To evaluate PRRT2 and β1–β4-subunit interactions, HEK293 cells were transfected with either HA-tagged PRRT2 [9] or the unrelated bacterial alkaline phosphatase (BAP) as a control [16]. For competition assays, Na_V_1.2-expressing HEK293 cells were transfected with HA-tagged PRRT2 and incubated with FLAG-tagged β4-subunit expressed in wild-type cells. Alternatively, HA-tagged PRRT2 and FLAG-tagged β4-subunit were co-expressed in Na_V_1.2-expressing HEK293 cells. For biotinylation assays and electrophysiological experiments, Na_V_1.2-expressing HEK293 cells were transfected with empty vector (pkH3; Addgene), HA-tagged PRRT2, and/or FLAG-tagged β4-subunit. All transfections were conducted with Lipofectamine 2000 (Invitrogen) 48 h before the experiments according to the manufacturer’s protocol (1.5–2 × 10^5^ cells per wells were transfected with 1 μg of each cDNAs with 2 μl of Lipofectamine 2000).

### SDS-PAGE and Western Blotting

SDS-PAGE was performed according to Laemmli [29]. Samples heated to 50 °C for 5 min without boiling were run on SDS-PAGE polyacrylamide gels and blotted onto nitrocellulose membranes (Whatman). Blotted membranes were blocked for 1 h in 5% milk in Tris-buffered saline (10 mM Tris, 150 mM NaCl, pH 8.0) plus 0.1% Triton X-100 and incubated overnight at 4 °C with the appropriate primary antibody. Membranes were washed and incubated at room temperature for 1 h with peroxidase-conjugated anti-mouse (1:3000; BioRad, Hercules, CA) or anti-rabbit (1:3000; BioRad, Hercules, CA) antibodies. Bands were revealed with the ECL chemiluminescence detection system (Thermo Fisher Scientific). Immunoblots were quantified by densitometric analysis of the fluorograms (Quantity One software; Bio-Rad, Hercules, CA) obtained in the linear range of the emulsion response.

### Pull-Down Assays

Wild-type HEK293 cells or Na_V_1.2-expressing HEK293 cells were transfected as previously described. After 48 h, cells were harvested in lysis buffer (150 mM NaCl, 50 mM Tris, 1 mM EDTA and 1% Triton X-100 supplemented with protease inhibitor cocktail) and centrifuged at 10000 × g for 10 min at 4 °C. Kept an aliquot for the input sample, the supernatant was incubated with 50 μL of either anti-FLAG® M2 Affinity Gel or monoclonal anti-HA-agarose affinity beads (Sigma-Aldrich) at 4 °C for 2 h. After extensive washes in lysis buffer and detergent-free lysis buffer, samples were resolved by sodium dodecyl sulfate–polyacrylamide gel electrophoresis (SDS-PAGE) and subjected to western blotting with anti-panNa_V_ (1:300; Sigma-Aldrich), anti-FLAG (1:2000; Sigma-Aldrich), or anti-HA (1:1000; Thermo Fisher Scientific) specific antibodies.

### Surface Biotinylation Assays

Forty-eight hours after transfection, Na_V_1.2-expressing HEK293 cells were incubated with 1 mg/ml of EZ-Link™ Sulfo-NHS-LC-Biotin (Thermo Fisher Scientific) in cold phosphate-buffered saline (PBS) for 35 min at 4 °C, with constant mixing. Free biotin was quenched, twice with 50 mM Tris pH 8, and once with cold PBS to remove the excess of biotin. Cells were then lysed in lysis buffer (150 mM NaCl, 50 mM Tris, 1 mM EDTA, and 1% Triton X-100) supplemented with protease inhibitor cocktail (Cell Signaling). Total cell lysates were centrifuged at 10000 × g at 4 °C for 10 min. Kept an aliquot for the input sample, the supernatant fraction was incubated with 150 µl of NeutrAvidin-conjugated agarose beads (Thermo Fisher Scientific) at 4 °C for 3 h. After extensive washes of the beads, samples were eluted, resolved by SDS-PAGE, and subjected to western blotting with anti-panNaV (1:300; Sigma-Aldrich), anti-Na/K ATPase pump 1 (1:1000; Merck), anti-FLAG (1:2000; Sigma Aldrich), or HA (1:1000; Thermo Fisher Scientific) antibodies.

### Electrophysiological Recordings and Analysis

Whole-cell voltage-clamp experiments were performed on HEK293 stably expressing Na_V_1.2 α-subunit transiently transfected with cDNA of empty vector pKH3 (MOCK), PRRT2-HA, β4-subunit, or PRRT2-HA + β4-subunit. Transfection was done with Lipofectamine 2000 as described above. To obtain better clamp control of cell under recording, isolated cells were used for electrophysiological experiments. Thus, transfected cells were enzymatically dissociated, replated at low density about 24 h post-transfection. All recordings were performed 24 h after replating; transfected cells were identified by fluorescence of co-transfected tomato protein reporter (Clontech). Recording solutions were previously described [16]. Briefly, the standard external solution contained the following (in mM): 140 NaCl, 3 KCl, 1 MgCl_2_, 1 CaCl_2_, 10 HEPES, 10 mannitol (pH 7.3 with NaOH); the standard internal solution contained  the following (in mM): 140 CsCl, 10 NaCl, 2 EGTA, 10 HEPES (pH 7.3 with CsOH). To induce resurgent currents, 200 μM Na_V_ β4 peptide (KKLITFILKKTREK-OH; Proteogenix), corresponding the COOH-terminal tail of full-length β4-subunit, was included in the pipette solution. Patch pipettes, prepared from thin-borosilicate glass (Hilgenberg), were pulled and fire-polished to a final resistance of 2–3 MΩ when filled with standard internal solution. Electrophysiological experiments were done using an EPC-10 amplifier (HEKA Electronik). Whole-cell voltage-clamp recordings of Na_V_ currents were acquired at 20 kHz and low-pass filtered at 4 kHz. Recordings with leak currents > 200 pA or series resistance > 10 MΩ were discarded. Data acquisition was performed using the PatchMaster program (HEKA Elektronik GmbH). Series resistance was compensated 80% (2 μs response time) and the compensation was readjusted before each stimulation. The membrane potentials in whole-cell recordings were uncorrected for Donnan liquid junction potentials of ~ 9 mV [30]. All experiments were performed at room temperature (22–24 °C). Whole-cell family currents of fast inactivating Na_V_ channels were evoked by 5 mV step depolarization (100 ms in duration) from − 80 to 65 mV and cells were held at a holding potential (V_h_) of − 120 mV. Steady-state inactivation curves were constructed by recording the peak current amplitude evoked by 20-ms test pulses to − 10 mV after 500-ms pre-pulses to potentials over the range of − 130 to 10 mV (V_h_ = − 120 mV). The conductance-voltage (G-V) curves were obtained by converting the maximal current values, evoked with a voltage step protocol in each cell, to conductance according to the extended Ohm’s law: G = I_Na_/(V − E_Na_), where I_Na_ is the peak Na^+^ current measured at potential V, and E_Na_ is the calculated Nernst equilibrium potential. G-V curves were normalized and fitted with the Boltzmann function G/G_max_ = 1/(1 + exp[(V − V_1/2_)/k]), where G is the conductance, G_max_ is the maximal conductance, V_1/2_ is the half-maximal voltage of activation, and k is the slope factor. Inactivation curves were fitted with the Boltzmann equation in the following form: 1/[1 + exp(V_1/2_ − V)/k]. Time-dependent rate of recovery from inactivation was calculated by pre-pulsing the cell with a 20-ms step to − 20 mV to inactivate the channels and then bringing back the potential to − 100 mV for increasing recovery durations (0.5, 1, 2, 4, 8, 32, 64, 128, 148 ms) before the test pulse of − 20 mV (V_h_ = − 120 mV). Time constants for recovery from inactivation were obtained by fitting data from each cell to a first order exponential function and averaging time constants across cells. Persistent currents were evoked from V_h_ = − 120 mV by 5-mV depolarization steps of 600 ms from − 60 to 50 mV. Resurgent currents were evoked with depolarization steps from V_h_ of − 120 mV to 30 mV (20 ms) to open channels, allowing them to undergo open-channel block, and subsequently repolarizing to potentials ranging from − 50 to 20 mV (60 ms) to allow the blocker to unbind. Persistent currents were measured in the last 60 ms of a 600 ms depolarizing step pulse. Resurgent currents were measured after 2.5 ms into the repolarization step to bypass fast tail currents. The percentage of persistent/resurgent currents were calculated by dividing the current amplitude by the peak transient current in each recorded cell. For currents of small amplitude, such as in persistent current recordings, 3–5 sweeps were recorded for each condition and averaged to improve the signal-to-noise ratio. To minimize space-clamp problems, we selected only isolated cells with a soma diameter of about < 30 µm for recordings. Membrane capacitance artifacts and leakage currents were eliminated by P/N leak subtraction procedure.

### Statistical Analysis

All data points are presented as mean ± standard error of the mean (sem) for number of cells or number of independent experiments (*n*), as detailed in the figure legends. Normal distribution of data was assessed using D’Agostino-Pearson’s normality test. The *F* test was used to compare variance between two sample groups. To compare two normally distributed sample groups, Student’s unpaired *t*-test was used. To compare two sample groups that were not normally distributed, the non-parametric Mann–Whitney’s *U*-test was used. To compare more than two normally distributed sample groups, we used one-way ANOVA, followed by either Bonferroni’s or Fisher’s test. In cases in which data were not normally distributed, non-parametric one-way ANOVA (Kruskal–Wallis’ test) was used, followed by the Dunn’s multiple comparison test. Alpha levels for all tests were 0.05% (95% CIs). Statistical analysis was conducted using the OriginPro-8 (OriginLab) and Prism (GraphPad Software) software.

## Results

### PRRT2 and the Na_V_ β4-Subunit Do Not Interact with Each Other or Compete for Binding to Na_V_1.2

Na_V_ β-subunits are known to positively modulate the Na^+^ channel membrane exposure and biophysical properties. Thus, it was of interest to investigate whether the inhibitory constraint of PRRT2 on Na^+^ channel activity was attributable to PRRT2-induced sequestration of β-subunits or interference with the binding of β-subunits to the Na_V_ α-subunit. To this aim, we performed affinity binding assays to assess the interactions between Na_V_1.2, β4-subunits and PRRT2. Among β-subunits, β4 was chosen for its high expression in the cerebellum [28], which is the main brain region responsible for the phenotype in PRRT2 KO mice and where PRRT2 reaches the highest expression under physiological conditions [8, 19]. Moreover, of the four β-subunits, β4 is the only subunit that enables the resurgent Na^+^ current responsible for high-frequency firing in neurons [31]. HA-tagged PRRT2 and β-subunits were transiently expressed in HEK293 cell clones stably transfected with human Na_V_1.2 and the PRRT2/Na_V_1.2 α/β complexes were pulled down with anti-HA beads and identified by western blotting with anti-Na_V_ α- and β-subunit antibodies.

**Fig. 1:**
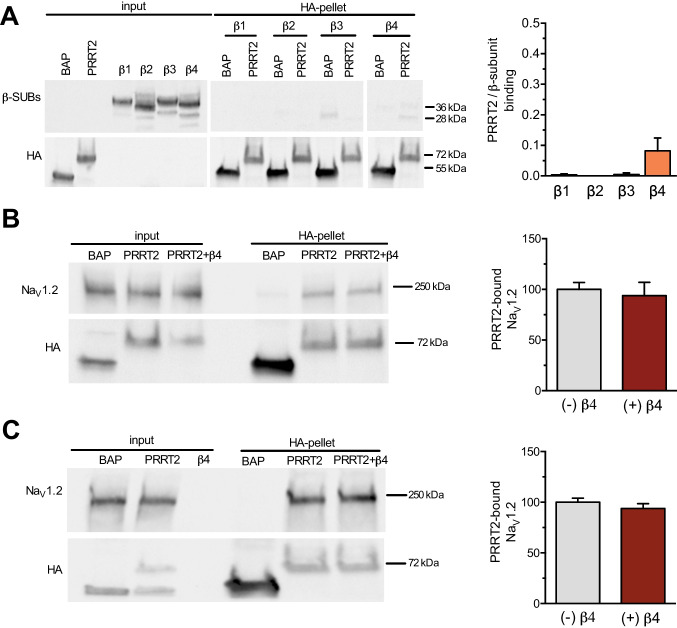
PRRT2 and the Na_V_ β4-subunit do not interact or compete for binding to Na_V_1.2. **A** *Left*: representative immunoblot of co-immunoprecipitation of β1/β4-subunits by PRRT2. HA-tagged PRRT2 (PRRT2), bacterial alkaline phosphatase (BAP), and FLAG-tagged β1/β4-subunits were expressed in wild-type HEK293 cells. Cell lysates (INPUT, 10 µg protein) and samples immunoprecipitated by anti-HA beads (HA-pellet) were analyzed by western blotting with anti-FLAG and anti-HA antibodies. The representative blots were cut from the same gel. *Right*: quantification of the FLAG immunoreactive signal in PRRT2 immunoprecipitates normalized to the control BAP values. Means ± sem of *n* = 3 independent experiments. **B** *Left*: representative immunoblot of co-immunoprecipitation of PRRT2 and Na_V_1.2 from extracts of Na_V_1.2-expressing stable HEK293 clones transiently transfected with either HA-tagged PRRT2 alone or with HA-PRRT2 + β4 subunit. BAP was used as a control. Cell lysates (INPUT, 10 µg protein) and samples immunoprecipitated by anti-HA beads (HA-pellet) were analyzed by western blotting with anti-panNa_V_ and anti-HA antibodies. *Right*: quantification of the Na_V_ immunoreactivity in PRRT2 immunoprecipitates expressed as ratios between normalized Na_V_1.2 and PRRT2 immunoreactivities. Means ± sem of *n* = 3 independent experiments. **C** *Left*: representative immunoblot of co-immunoprecipitation of Na_V_1.2 by PRRT2 in the presence or absence of an excess of β4-subunit. HA-tagged PRRT2 or BAP was transfected in Na_V_1.2-expressing HEK293 cells, whereas the β4-subunit (β4) was overexpressed in wild-type HEK293 cells. The extract from the β4-subunit expressing cells was added to the HA-immunoprecipitated PRRT2/Nav 1.2 complex. Cell lysates (INPUT, 10 µg protein) and samples immunoprecipitated by anti-HA beads (HA-pellet) were analyzed by western blotting with anti-panNa_V_ and anti-HA antibodies. *Right*: quantification of the Na_V_ immunoreactivity in PRRT2 immunoprecipitates normalized to the BAP values. Means ± sem of *n* = 3 independent experiments.

We asked whether PRRT2 can bind directly to the cytosolic or transmembrane domains of β-subunits, thus decreasing their availability for the Na_V_1.2 α/β complex. Affinity binding assays performed in wild-type HEK293 cells expressing HA-tagged PRRT2 and either the negative control BAP or the various β-subunits revealed that PRRT2, pulled down by anti-HA beads, did not significantly associate with either β-subunit detected by western blotting with β1/β4-subunit specific antibodies (Fig. 1A). Next, using the Na_V_1.2 α-subunit as a hub, we investigated whether PRRT2 and the β4-subunit can bind to the Na_V_1.2 α-subunit independently at distinct sites or they compete for a single association site. When both HA-tagged PRRT2 and β4-subunit were expressed in HEK293 cell clones stably transfected with human Na_V_1.2, pull down of PRRT2 with anti-HA beads resulted in the co-precipitation of similar amounts of Na_V_1.2 α-subunit together with detectable amounts of β4-subunit (Fig. 1B). To assay for a direct competition of PRRT2 and β4-subunit for a shared binding site on the α-subunit, HA-tagged PRRT2 or BAP was expressed in HEK293 cells stably transfected with human Na_V_1.2 and the HA-PRRT2/Na_V_1.2 immunoprecipitated complex was challenged in the absence or presence of an excess of β4-subunit expressed in wild-type HEK293 cells. Also under these conditions, the presence of an excess β4-subunit did not inhibit the binding of PRRT2 to Na_V_1.2, as the amount of α-subunit present in the immunocomplexes was not affected in the presence of absence of β4-subunit, as shown by western blotting with anti-Na_V_ antibodies (Fig. 1C).

To ascertain whether other β-subunits bind to the Na_V_1.2 α-subunit independently of PRRT2, we tested the β3 that, opposite to β4, is non-covalently associated and binds to different regions of the α-subunit. We found a very similar behavior in pulldown competition assays with PRRT2, indicating that the findings can be extended to the other β-subunits (Supplementary Fig. 1).

Taken together, the data indicate that PRRT2 and the Na_V_ β-subunits do not interact with each other and bind independently to the Na_V_1.2 α-subunit.

### PRRT2 and Na_V_ β4-Subunit Have Opposite Actions of the Trafficking of the Na_V_1.2 α-Subunit to the Plasma Membrane

**Fig. 2:**
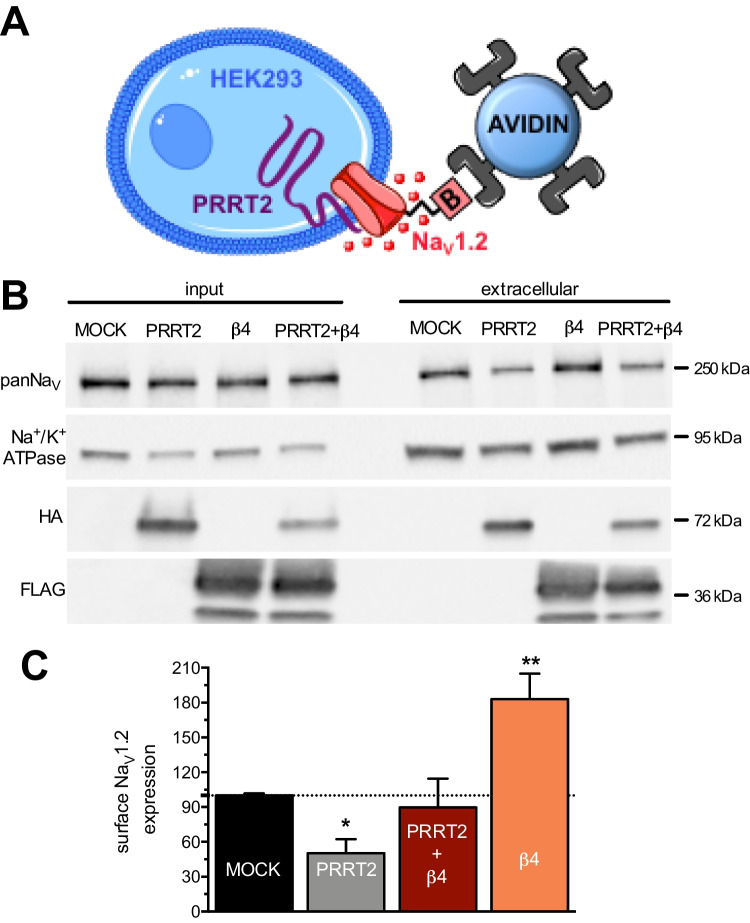
PRRT2 and the Na_V_ β4-subunit have opposite effects on membrane targeting and exposure of Na_V_1.2 channels. **A** Schematics of the biotinylation experiment. **B** Representative immunoblots of cell surface biotinylation performed in HEK293 cells expressing Na_V_1.2 and transfected with empty vector (MOCK), PRRT2-HA, β4-FLAG, or both. Total lysates (input; *left*) and biotinylated (cell surface; *right*) fractions were analyzed by western blotting. Membranes were probed with antibodies to panNa_V_, HA, FLAG, and Na/K-ATPase (Na/K), with the latter used as marker of cell surface fractions*.* **C** The cell surface Na_V_ immunoreactivity is expressed in percent of the control MOCK value after normalization to Na/K-ATPase immunoreactivity. Means ± sem of *n* = 4 independent experiments. Two-way ANOVA revealed no significant interaction between PRRT2 and β4-subunit (*F*_1,12_ = 3.238; *p* = 0.1). *p < 0.05, **p < 0.01; one-way ANOVA/Fisher’s least significant difference tests versus MOCK

It is well known that the Na_V_ β-subunits enhance the trafficking of the α-subunits to the plasma membrane, resulting in a larger population of active channels exposed to the extracellular *milieu* [28]. On the other hand, we recently shown that PRRT2 plays the opposite action of inhibiting the membrane exposure of Na_V_1.2/Na_V_1.6 that causes a decrease in Na^+^ current [16]. Thus, we investigated the effect of the simultaneous presence of both α-subunit modulators on the surface exposure of the channel. To this aim, we performed surface biotinylation of HEK293 cell clones stably expressing the human Na_V_1.2 α-subunit that had been transiently transfected with HA-tagged PRRT2, β4-subunit or both and quantified the amount of surface-labeled Na_V_1.2 α-subunits. The results show that, while PRRT2 and β4-subunit have the reported opposite actions on the exposure of Na_V_1.2 channels on the plasma membrane, the combined expression of the two modulators was not significantly different from the control sample, indicating that the opposite actions of PRRT2 and β4-subunit on Na_V_1.2 channel trafficking occur independently and are simply additive (Fig. 2).

### PRRT2 Expression Counteracts the Increase in Na_V_1.2 Current Density Induced by β4-Subunit

**Fig. 3:**
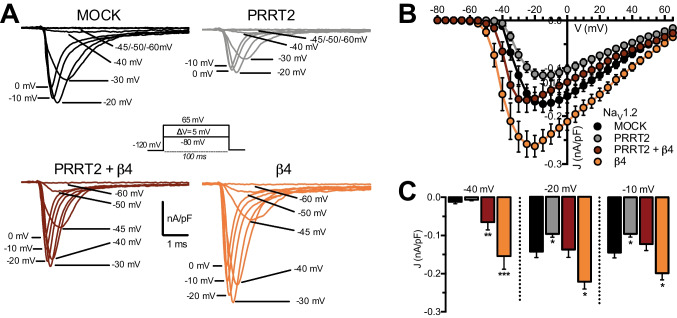
PRRT2 and the β4-subunit have opposite effects on the expression of the Na_V_1.2 transient current. **A** Representative whole-cell transient Na^+^ currents recorded in HEK293 cells stably expressing Na_V_1.2 and transiently transfected with empty vector (MOCK, black), PRRT2 (gray), β4-subunit (orange), or PRRT2 + β4 subunit (dark red). Currents were elicited by a protocol (*inset*) consisting of 5-mV depolarization steps from − 80 to 65 mV from a holding potential of − 120 mV. For clarity, the first 6 ms of the 100-ms steps for eight representative traces per condition are plotted. **B**, **C** Current density (*J*) versus voltage (*V*) relationship (**B**) for all the studied experimental conditions. The statistical analysis of *J* values at three representative voltages (− 40/− 20/− 10 mV) is reported (**C**). Data are expressed as means ± sem (MOCK, *n* = 36; PRRT2, *n* = 22; β4, *n* = 18; PRRT2 + β4, *n* = 17). Two-way ANOVA revealed no significant interaction between PRRT2 and β4-subunit on the amplitude of the macroscopic Na^+^ current (− 40 mV: *F*_1,88_ = 0.021; *p* = 0.88; − 20 mV: *F*_1,90_ = 1.2; *p* = 0.276; − 10 mV: *F*_1,89_ = 0,804; *p* = 0.37). *p < 0.05, **p < 0.01, ***p < 0.001 versus MOCK, Kruskal–Wallis/Dunn’s tests

To test the influence of PRRT2 on the properties of Na^+^ currents mediated by the physiological complex Na_V_ α-subunit-β4-subunit, macroscopic whole-cell currents were recorded from HEK293 cells stably expressing Na_V_1.2 which had been sequentially transfected with empty vector (pKH3; MOCK), PRRT2, β4-subunit or with PRRT2 + β4-subunit (Fig. 3A). Families of transient Na^+^ currents were elicited by applying 100-ms depolarizing steps ranging from − 80 mV to 65 mV from a holding potential of − 120 mV (*inset*). Current density (J)/voltage (V) curves were built for all experimental conditions by normalizing the peak current at each voltage by the cell capacitance (Fig. 3B). When compared to control cells, PRRT2-expressing cells showed the previously reported reduction of the transient Na^+^ current across a wide voltage range and in the absence of voltage shifts in the peak Na^+^ current [16] (Fig. 3B, C). As expected, expression of the β4-subunit had a positive modulatory effect on Na^+^ current density with a two-fold increase of the Na_V_1.2 current density, as compared to MOCK-transfected cells with a shift of the peak current toward more negative voltages (Fig. 3B, C). The observation that the properties of Na_V_1.2 currents were modified after either β4-subunit or PRRT2 transfection suggests that both these proteins were successfully integrated into the channel signaling complex and exerted their modulatory action.

Interestingly, when PRRT2 was co-expressed with the β4-subunit, it neutralized the positive modulation of the β-subunit on amplitude of the Na^+^ current density, while the β4-subunit-induced left shift of the J/V curve was preserved (Fig. 3B, C). The data confirm that PRRT2 and β4-subunit affect membrane targeting and exposure of the Na_V_1.2 α-subunit in opposite directions, indicating that they do that via independent mechanisms of action with resulting additive effects.

### PRRT2 and β4-Subunit Differentially Affect the Kinetics of Activation and Inactivation of Na_V_1.2 Channels

**Fig. 4:**
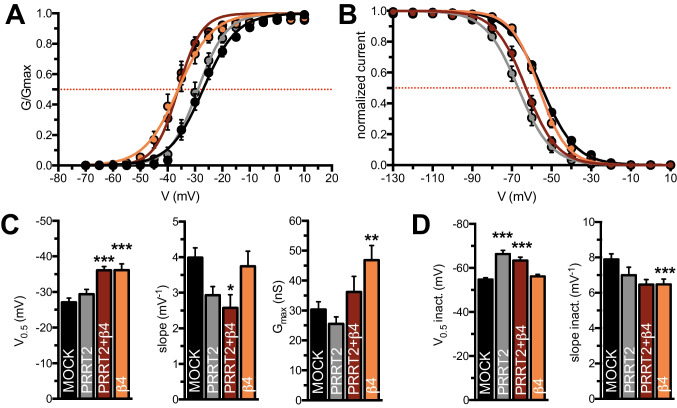
PRRT2 and β4-subunit differentially affect the kinetics of activation and inactivation of Na_V_1.2 channels. **A**, **B** Voltage dependence of activation (**A**) and steady-state inactivation (**B**) curves fit with a Boltzmann function for all conditions tested. Activation was studied using recordings obtained with the protocol depicted in Fig. 3A. Steady-state inactivation was obtained with a protocol in which the cell under study was held at a series of voltages ranging from − 130 mV to 30 mV for 500 ms followed by a 20-ms step pulse to − 10 mV to measure channel availability using a holding potential of − 120 mV. **C**, **D** Mean parameters of activation (**C**) and steady-state inactivation (**D**) curves fit to data obtained from all condition tested. All data are expressed as means ± sem. Activation: MOCK, *n* = 36; PRRT2, *n* = 22; β4, *n* = 18; PRRT2 + β4, *n* = 17. Steady-state inactivation: MOCK, *n* = 44; PRRT2, *n* = 16; β4, *n* = 28; PRRT2 + β4, *n* = 24. No significant differences were found in the activation kinetics between β4-subunit alone and PRRT2 + β4-subunit groups, as well as in the inactivation kinetics between PRRT2 alone and PRRT2 + β4-subunit groups. Two-way ANOVA revealed no significant interaction between PRRT2 and β4-subunit on the analyzed biophysical parameters (activation V_0.5_: *F*_1,89_ = 0.744, *p* = 0.39; G_max_: *F*_1,89_ = 0.645 *p* = 0.424; inactivation V_0.5_: *F*_1,108_ = 3.57, *p* = 0.061; inactivation slope: *F*_1,108_ = 1.510, *p* = 0.221). *p < 0.05, **p < 0.01, ***p < 0.001 versus MOCK; one-way ANOVA/Bonferroni’s tests or Kruskal–Wallis/Dunn’s tests

To dissect the effects of PRRT2 on the biophysical properties of Na_V_1.2 α-subunits in the presence of the β4-subunit, we next examined the voltage dependence of channel activation and steady-state inactivation. In a previous study [16], we observed that PRRT2 did not affect the Na_V_1.2 activation curves, while it favored channel inactivation at more negative potentials (Fig. 4A, B). On the other hand, the β4-subunit is known to cause a leftward shift of the activation curve of Na_V_1.2, without affecting the voltage dependence of inactivation [27].

The specific modulations of the Na_V_1.2 biophysical properties by PRRT2 and the β4-subunit were fully preserved when the two auxiliary proteins were co-expressed. Under this condition, the activation curves displayed the leftward shift as signature of the β4-subunit, while the steady-state inactivation curves were shifted at more negative potentials by the concomitant action of PRRT2 (Fig. 4A, B). Notably, β4 exerted its action on the activation irrespective of the absence or presence of PRRT2 and PRRT2 exerted its action on the inactivation irrespective of the absence or presence of β4.

The quantitation of the main biophysical parameters obtained from the Boltzmann fitting of individual activation and inactivation curves confirmed that the voltage of half-maximal activation (V_0.5_), slope and maximum conductance of activation (G_max_) were significantly changed in the presence of the β4-subunit and not affected by PRRT2 (Fig. 4C), while the voltage of half-maximal inactivation (V_0.5_) was decreased by PRRT2 and not affected by of the β4-subunit that only induced a slight decrease of the slope of inactivation (Fig. 4D).

### PRRT2, but not the β4-Subunit, Modulates the Na_V_1.2 Recovery Kinetics from Inactivation

**Fig. 5:**
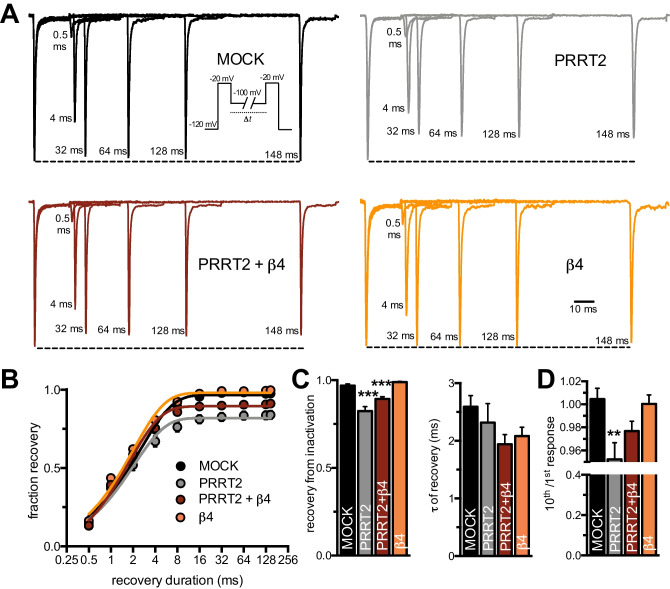
PRRT2, but not the β4-subunit, modulates the Na_V_1.2 recovery kinetics from fast inactivation. **A** Representative channel recovery from inactivation current traces recorded for all the experimental conditions. Recordings were obtained pre-pulsing cells to − 20 mV for 20 ms to inactivate Na^+^ currents and then coming back to a recovery potential of − 100 mV for increasing durations before the repetition of test pulse to − 20 mV. For clarity, 6 of the nine time intervals are shown. The dotted line represents the first pulse peak amplitude. **B** Time courses of the recovery from inactivation of peak currents at − 100 mV for all condition studied are plotted on a semi-logarithmic scale. **C** Mean (± sem) values of plateau and *τ* of recovery estimated from one-phase decay fit to the data. **D** Relationship between the 10th and the 1st test pulse evoked by protocol displayed in **A**. All data are expressed as means ± sem (MOCK, *n* = 46; PRRT2, *n* = 25; PRRT2 + β4, *n* = 28; β4, *n* = 29). Two-way ANOVA revealed no significant differences were found between PRRT2 alone and PRRT2 + β4-subunit groups (plateau of recovery: *F*_1,124_ = 3.286, *p* = 0.072; *τ* of recovery: *F*_1,124_ = 0.09, *p* = 0.764; 10th/1st response: *F*_1,124_ = 1.86, *p* = 0.174). **p < 0.01, ***p < 0.001 versus MOCK; Kruskal–Wallis/Dunn’s tests

We measured channel recovery from inactivation using a voltage command protocol in which we evaluated the peak current by: (i) measuring current evoked by a first depolarizing step to − 20 mV; (ii) allowing current to recover from inactivation at − 100 mV for progressively increasing time intervals; (iii) measuring current recovery with a second test pulse to − 20 mV (Fig. 5A, *inset*). When compared to MOCK-transfected cells, the extent of channel recovery was significantly decreased by PRRT2, while it was unaffected by the β4-subunit. When both auxiliary proteins were co-expressed, the PRRT2-induced decrease of the recovery plateau was only slightly attenuated, but still significant with respect to MOCK-transfected cells, while the time constant of recovery was not significantly modified under the various experimental conditions (Fig. 5B, C). The PRRT2-induced incomplete Na_V_1.2 recovery, previously observed by Fruscione et al*.* [16], is likely attributable to an effect of PRRT2 on the slow inactivation of the channel.

Moreover, the β4-subunit expression reduces the use-dependent inhibition of Na_V_1.2 by PRRT2 as shown by the ratio between the current evoked by the last and first step of the protocol used to study the recovery from fast channel inactivation (Fig. 5D). All these results indicate that PRRT2 and β4-subunit operate together into the Na_V_1.2 channel signaling complex by producing discrete and distinct modulations of the channel biophysics.

### PPRT2 and β4-Subunit Independently Modulate the Na_V_1.2 Persistent and Resurgent Currents

**Fig. 6:**
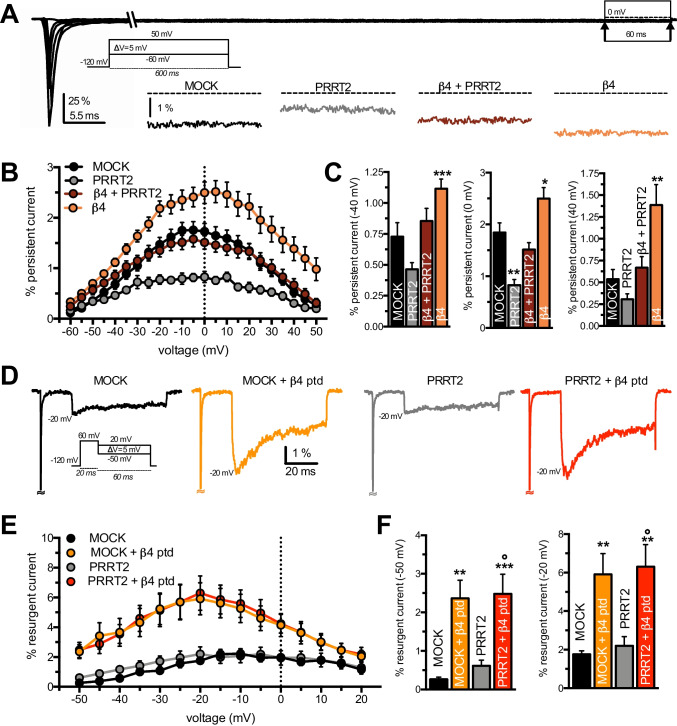
PRRT2 and β4-subunit independently modulate the Na_V_1.2 persistent and resurgent currents. **A** Representative MOCK persistent Na_V_1.2 currents evoked by depolarizing steps from − 60 to 50 mV with 5-mV increments, lasting 600 ms (*inset*) in MOCK-transfected Na_V_1.2-expressing HEK293. For clarity, only currents evoked at − 60, − 40, − 20, 0, 20, and 40 mV are reported. The highlighted box at the end of stimulation indicates the region of the trace in which the persistent current was measured. The insets show zoomed views of the maximal persistent current for all conditions tested. **B** Persistent current, measured as the mean current in the last 60 ms of each 600 ms step and normalized to the transient current peak, is plotted versus voltage in each cell. **C** Bar plots of the mean (± sem) values of the normalized persistent current amplitude recorded at three distinct voltages (− 40, 0, and 40 mV) for all tested conditions. Data are expressed as means ± sem (MOCK, *n* = 24; PRRT2, *n* = 10; PRRT2 + β4, *n* = 20; β4, *n* = 21). **D** Representative peak resurgent current traces generated by Na_V_1.2-expressing HEK293 cells either mock-transfected or transfected with PRRT2 recorded in the presence (yellow/red) or absence (black/gray) of the β4 COOH-terminal peptide (β4 ptd) in the intracellular recording solution. Currents were evoked with a family of steps depolarizations from − 120 mV to 30 mV for 20 ms to open the channels, allow them to undergo open-channel block, and subsequently repolarize to a different potential ranging from − 50 mV to 20 mV for 60 ms to allow the blocker to unbind. For clarity, only the peak resurgent current trace for all condition is plotted. **E** Resurgent currents evoked during repolarization were normalized to peak transient current at each voltage for all experimental conditions. Peak resurgent current was measured for each voltage after 2.5 ms into the repolarization step to bypass fast tail currents. **F** Bar plots of the mean (± sem) values of the normalized resurgent current amplitude recorded at three distinct voltages at − 20 and − 50 mV for all conditions tested. All data are expressed as means ± sem (MOCK, *n* = 6; MOCK + β4 ptd, *n* = 12; PRRT2, *n* = 6; PRRT2 + β4 ptd, *n* = 14). Two-way ANOVA revealed no significant interaction between PRRT2 and β4-subunit on the amplitude of both the persistent and the resurgent Na^+^ currents (persistent at: − 40 mV, *F*_1,71_ = 0.002, *p* = 0.988; 0 mV: *F*_1,71_ = 0.00519, *p* = 0.942; 40 mV, *F*_1,71_ = 1.94, *p* = 0.167; resurgent at: − 50 mV, *F*_1,34_ = 0.047, *p* = 0.828; − 20 mV: *F*_1,34_ = 0.00043, *p* = 0.983). *p < 0.05, **p < 0.01, ***p < 0.001, versus MOCK; one-way ANOVA/Bonferroni or Kruskal–Wallis/Dunn tests

Increases in both resurgent and persistent currents through Na_V_ channels are associated with neuronal hyperexcitability and increase in firing rates [32, 33]. Thus, we analyzed the effects of the combined expression of PRRT2 and β4-subunit on these currents evoked in Na_V_1.2-expressing HEK293 cells using specific protocols. Since the persistent Na^+^ current is a non-inactivating (or very slowly inactivating) current, we measured it over the last 60 ms of the 600 ms incremental conditioning steps for each condition tested (Fig. 6A, *inset*). Due to the variability of current density across cells, the persistent current amplitude was normalized to the peak amplitude of the transient current for each cell, and the percentage of persistent current was plotted *versus* the applied voltage. We found that when PRRT2 and β4-subunit were individually expressed, the percent amplitude of the Na_V_1.2 persistent current was decreased by PRRT2 and increased by the β4-subunit, when compared to MOCK-transfected cells. When both auxiliary proteins were co-expressed, the amount of persistent current was similar that recorded in MOCK-transfected cells (Fig. 6B, C). These findings, which closely resemble the behavior of the macroscopic transient Na^+^ current (see Fig. 3), demonstrate that PRRT2 and β4-subunit modulate independently the Na_V_1.2 persistent current when expressed in the same channel complex and that their effects are purely additive.

The resurgent Na^+^ current is caused by the influx of Na^+^ ions through Na_V_ channels during repolarization [34]. To elicit that, we applied an initial depolarizing step from − 120 to 60 mV, followed by subsequent incremental repolarizing steps from − 50 mV to 20 mV (Fig. 6D, *inset*). Because the expression of the full-length β4-subunit is not sufficient to produce resurgent currents in Na_V_1.2-expressing HEK293 cells, we included in the pipette solution a β4 peptide (β4-ptd) derived from the COOH-terminal of the β4-subunit (see Materials and Methods), that is known to induce resurgent currents [28, 35, 36].

Then we recorded Na^+^ resurgent currents in Na_V_1.2-expressing HEK293 cells which had been transfected either with the empty vector or with PRRT2 cDNA in the absence or presence of the β4-peptide in the pipette solution (MOCK and MOCK + β4-ptd, respectively) (Fig. 6D). Also in this case, to reduce the variability in current density across recorded cells, the resurgent current at each repolarizing voltage step was normalized to the peak amplitude of the transient current recorded in the same cell under the same experimental condition. While, as expected, the presence of β4-peptide greatly increased the resurgent current in a wide range of voltages, PRRT2 was totally ineffective in modulating the resurgent current in MOCK-transfected cells in both presence and absence of the β4-peptide (Fig. 6E, F). Hence, these data it appears that the expression and modulation of the Na_V_1.2 persistent current depends exclusively on the presence of the β4-peptide and is independent of PRRT2.

## Discussion

### PRRT2 and β-Subunits Are Both Na_V_ Modulatory Proteins

The neuron specific PRRT2 is an important modulator of presynaptic functions and intrinsic excitability. Neuronal circuits lacking PRRT2 become hyperexcitable [37]. Accordingly, patients bearing loss-of-function mutations in the *PRRT2* gene or PRRT2 KO mice are affected by paroxysmal manifestations, whose pleiotropism ranges from paroxysmal kinesigenic dyskinesia to epilepsy and migraine. Based on previous studies, these phenotypes result from a mixed synaptopathy/channelopathy [3, 38]. Indeed, we demonstrated that PRRT2 acts, in murine and human neurons, as a negative modulator of Na_V_1.2/Na_V_1.6 channels, without affecting the Na_V_1.1 subtype that is essential for the excitability of inhibitory neurons [16]. These findings well correlate with the therapeutic efficacy of Na_V_ channel inhibitors in ameliorating the clinical phenotype of PRRT2-linked disorders [22, 25, 36]. PRRT2 decreases the membrane targeting and exposure of active Na_V_ channels on the plasma membrane and, in addition, it modulates their biophysical properties by shifting to the left the inactivation curve and decreasing channel recovery from inactivation [16]. PRRT2 can thus be envisaged as a novel inhibitory auxiliary subunit of the Na_V_1.2/Na_V_1.6 α-subunits.

The best-known auxiliary subunits modulating both membrane exposure and kinetics of the pore-forming Na_V_ α-subunit are the β subunits [β1-β4] that, similarly to PRRT2, are single membrane spanning domain proteins. Among β-subunits, β4 was chosen for being highly expressed in the cerebellum [28], the main brain region responsible for the PRRT2 loss-of-function phenotype [8, 19] and the only subunit that enables the resurgent Na^+^ current [31]. When expressed in heterologous systems, the β4 subunit increases the Na^+^ current density by enhancing the membrane exposure of Na_V_ α-subunits, shifts the voltage dependence of channel activation toward more negative voltages and accelerates the rate of activation [27, 28, 39].

### PRRT2 and β4 Exert Independent and Distinct Modulations of Density and Biophysical Properties of Na_V_1.2

Given that both PRRT2 and β subunits associate with the pore-forming α-subunit but exert opposite effects on its membrane exposure and biophysical properties, we considered the possibility that PRRT2 and β subunits interfere with each other in binding to the α-subunit, or they act independently as auxiliary subunits on the common Na_V_ target. It has been shown that β2 and β4 subunits covalently interact with Na_V_ α-subunit by forming an extracellular disulfide bond [40, 41]. While direct competition by PRRT2 at this site it is not possible due to the substantial lack of an extracellular domain [9], other potential α/β interaction sites are possible. In fact, interactions between the transmembrane domain of Na_V_1.4 α-subunit [42] and modulation of Na_V_ biophysical properties by the intracellular domains of β2 and β4 [43, 44] have been reported, suggesting the existence of other potential interaction sites in the transmembrane/intracellular domains of α and β Na_V_ subunits. Therefore, it was important to check a possible PRRT2/β competition in these regions of the channel complex.

Looking at the molecular interactions within the Na_V_1.2 supramolecular complex, we found that: (i) both PRRT2 and β subunits bind to Na_V_1.2 channels; (ii) PRRT2 does not interact with any of the β subunits; (iii) β subunits do not compete for PRRT2 binding to Na_V_1.2 channels; (iv) PRRT2 and β subunits have opposite and additive effects on the targeting and membrane exposure of Na_V_1.2 channels. These data indicate that PRRT2 and β4 have distinct docking sites and bind independently to Na_V_1.2 α-subunit, without competing for a common site, resulting in a purely additive effect on the membrane targeting and density of the Na_V_1.2 channel.

**Fig. 7:**
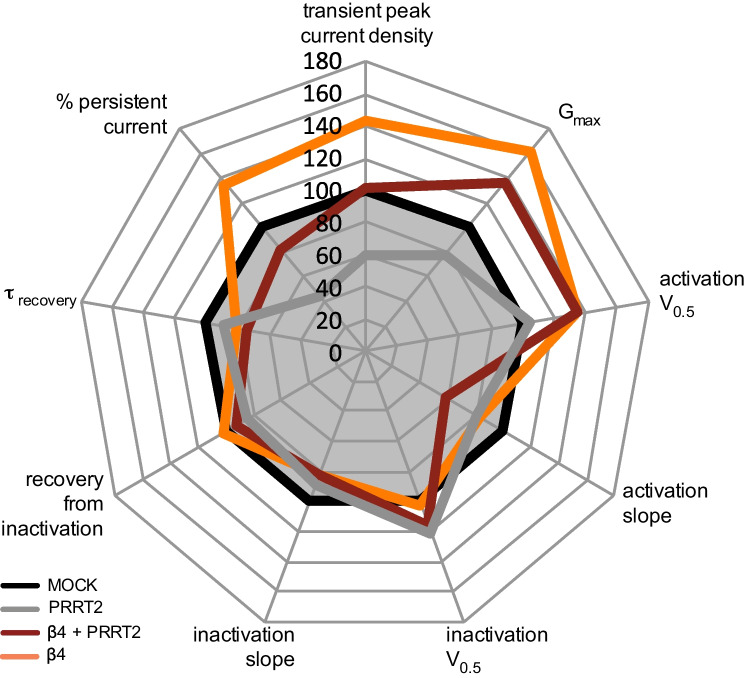
Summary of the differential modulation of Na_V_1.2 channels by PRRT2 and the β4-subunit. Radar plot showing the effects of the single or combined expression of PRRT2 and Na_V_ β4-subunit on Na_V_1.2 properties. The plot was built with each spoke arranged in such a way that points lying outside the control condition (MOCK plot; black) are indicative of a gain-of-function, while points lying within the MOCK plot indicate a loss-of-function. All parameters are expressed in percent of the MOCK group. Values of transient peak current density were recorded at − 20 mV. The percentage values of persistent current were obtained from recordings performed at 0 mV

The subsequent electrophysiological investigation revealed that PRRT2 and β4 have effects that are partly opposite and partly distinct and complementary on current density and biophysics. The effects of the single and combined expression of the two auxiliary proteins are schematically summarized in the radar plot of Fig. 7. The β4 subunit increases the transient Na^+^ current; PRRT2 only decreases the Na^+^ current; the PRRT2/β4 association displays a pure additive effect whereby the Na^+^ current remains unchanged but shifted to the left due to the specific β4 effect. PRRT2 does not affect the kinetics of activation; β4 induces a marked shift of the activation curve to negative voltages and an increase in G_max_ that is maintained unaffected in the presence of PRRT2. On the opposite, β4 is ineffective on the kinetics of inactivation and recovery from inactivation; PRRT2 shifts to negative voltages the kinetics of inactivation and decreases the recovery from inactivation, effects that remain unaltered in the presence of β4. Overall, these data demonstrate that the two proteins cover distinct, partly overlapping, domains of Na_V_ channel modulation, but that their effects on the Na^+^ channel biophysical properties are purely additive and likely depend on the degree of neuron-specific expression and turnover of the respective auxiliary proteins.

The effects on the persistent Na^+^ current reproduced those observed on the transient current, with β4 increasing the current amplitude and PRRT2, as recently reported in cerebellar granule cells [17], decreasing it, so that the two opposite effects were totally neutralized in the presence of both proteins. On the other hand, the resurgent Na^+^ current generated in the presence of the intracellular β4 peptide was specifically increased by β4, irrespective of the presence or absence of PRRT2 that was previously shown not to modulate this current in cerebellar granule cells [17].

### Push–Pull Control of Na_V_ Function by PRRT2 and β-Subunits

These results open the possibility that PRRT2 and Na_V_ β-subunits are part of a push–pull mechanism that fine tunes the activity of Na^+^ channels and their targeting and exposure on the membrane. In addition, the two auxiliary proteins have specific and non-superimposable effects on the channel biophysical properties. The cell density of Na_V_ channels is the fundamental determinant of intrinsic excitability in neurons. In such a complex system, a dual control of Na^+^ channel density, particularly of the PRRT2-sensitive Na_V_1.2/Na_V_1.6 subtypes responsible for the excitability of excitatory neurons, is the most efficient way to control the “excitability tone” over time. Under this double control, the activity of Na_V_ α-subunits can be modulated by the relative expression of PRRT2 and β-subunits, thus determining the heterogeneity in intrinsic excitability observed in various neuronal populations.

The majority of the expressed Na_V_ α-subunits are intracellular, within the endoplasmic reticulum, Golgi apparatus and secretory vesicles [45]. Here, α-subunits undergo extensive glycosylation essential for the delivery of the channel to the plasma membrane, its stability and biophysical properties [46, 47]. Na_V_ β-subunits associate with the channel at the intracellular level and promote its trafficking to the plasma membrane [48, 49]. Experimental evidence suggests that this requires N-linked glycosylation β-subunits, as well as a positive effect of the β-subunits on glycosylation of the α-subunits that promotes stabilization of the channel at the plasma membrane [50, 51]. Moreover, β-subunits also belong to the CAM family of adhesion molecules, and cell adhesion and interactions with the cytoskeleton by β-subunits seem to be critical to the cell surface expression of α-subunit [52, 53].

A question that remains unanswered is how PRRT2 acts in inhibiting the Na_V_ density on the plasma membrane. In principle, PRRT2 can modulate the membrane surface density of Na_V_ channels through various potential mechanisms, namely (i) slowing down exocytosis of intracellular Na_V_-containing vesicles, (ii) enhancing the turnover of membrane domains containing Na_V_ channels, and (iii) modulating Na_V_ channel interactions with the cytoskeleton. Although no direct evidence is as yet available, interactions of PRRT2 with SNARE proteins responsible for membrane fusion at nerve terminals have been reported [5, 10, 11], as well as potential interactions of the proline-rich cytosolic N-terminal domain of PRRT2 with SH3-domain bearing proteins involved in endocytosis, such as endophilin and intersectin [9]. Moreover, PRRT2 has been recently shown to modulate the actin-based cytoskeleton and, when expressed in non-neuronal cell lines, inhibits cell motility and focal adhesion turnover [12, 13].

## Conclusions

The results indicate that PRRT2 can be considered a novel Na_V_ auxiliary subunit with specificity for the 1.2/1.6 channel subtypes. Although the results were obtained in a heterologous expression system, the previous data indicate that they may hold true also in native neurons. We demonstrate that PRRT2 and β-subunits do not interact; rather, they can act in concert by modulating density and properties of voltage-dependent Na^+^ channels. These effects may represent the basis of a homeostatic mechanism that controls the level of intrinsic neuronal excitability in a dynamic fashion based on specific combinations of PRRT2 and β-subunit expression.

## Supplementary Information

Below is the link to the electronic supplementary material.

Supplementary file1 (PDF 202 KB)

## Data Availability

The datasets generated during and/or analyzed during the current study are available from the corresponding authors on reasonable request.
