# Supplementary material for: A Push–Pull Mechanism Between PRRT2 and β4-subunit Differentially Regulates Membrane Exposure and Biophysical Properties of NaV1.2 Sodium Channels
Source: Mol Neurobiol. 2022 Nov 28;60(3):1281–96. doi: 10.1007/s12035-022-03112-x (PMC9899197; doi:10.1007/s12035-022-03112-x)
Supplement: Supplementary file 1 — Supplementary file1 (PDF 202 KB) [file 12035_2022_3112_MOESM1_ESM.pdf]

A PUSH-PULL MECHANISM BETWEEN PRRT2 AND β4 DIFFERENTIALLY REGULATES MEMBRANE EXPOSURE AND BIOPHYSICAL PROPERTIES OF NaV1.2 SODIUM CHANNELS

Pierluigi Valente[1,2]*, Antonella Marte[1,2], Francesca Franchi[1,3], Bruno Sterlini[1,3], Silvia Casagrande[1], Anna Corradi[1,2], Pietro Baldelli[1,2], Fabio Benfenati[2,3]*

[1] Department of Experimental Medicine, Section of Physiology, University of Genova, Viale Benedetto XV, 3, 16132 Genova, Italy.

[2] IRCCS, Ospedale Policlinico San Martino, Largo Rosanna Benzi 10, 16132 Genova, Italy.

[3] Center for Synaptic Neuroscience and Technology, Istituto Italiano di Tecnologia, Largo Rosanna Benzi 10, 16132 Genova, Italy.

* Corresponding authors

SUPPLEMENTARY INFORMATION

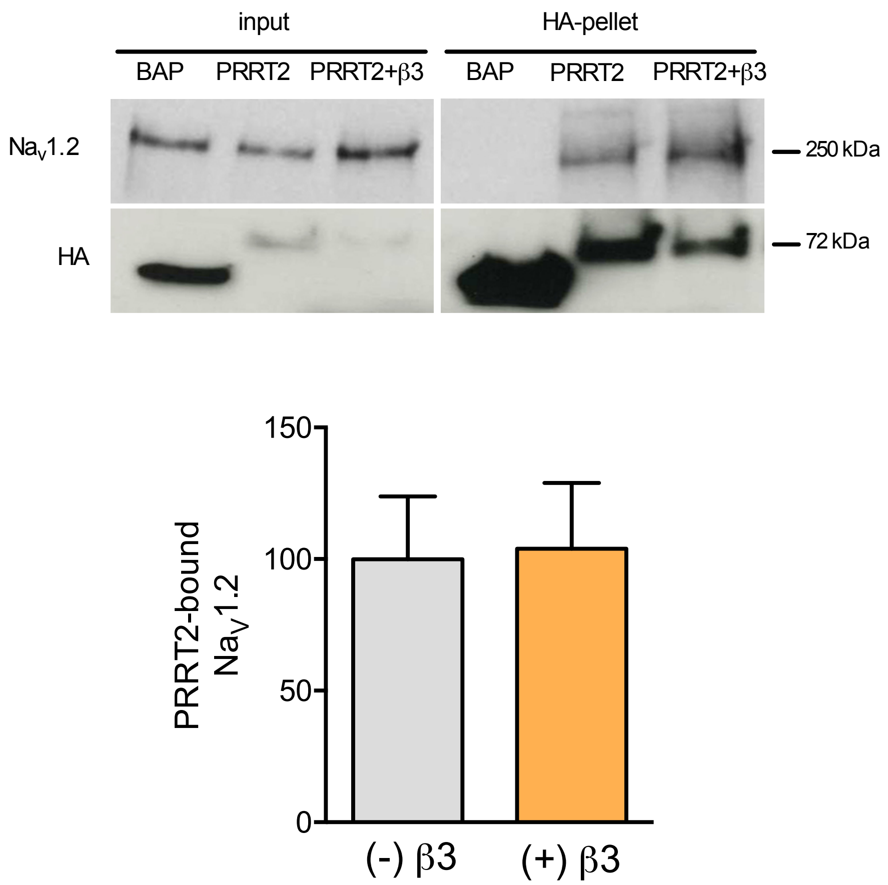

Supplementary Figure 1. PRRT2 and the NaV β3-subunit do not interact or compete for binding to NaV1.2.

A. *Upper panel*: Representative immunoblot of co-immunoprecipitation of PRRT2 and NaV1.2 from extracts of NaV1.2-expressing stable HEK293 clones transiently transfected with either HA-tagged PRRT2 alone or with HA-PRRT2 + β3 subunit (β3). BAP was used as a control. Cells lysates (INPUT, 10 µg protein) and samples immunoprecipitated by anti-HA beads (HA-pellet) were analyzed by western blotting with anti-panNaV and anti-HA antibodies. *Lower panel*: Quantification of the NaV immunoreactivity in PRRT2 immunoprecipitates expressed as ratios between normalized NaV1.2 and PRRT2 immunoreactivities. Means ± sem of n = 3 independent experiments.
